# Principal Component Analysis of Dynamic Relative Displacement Fields Estimated from MR Images

**DOI:** 10.1371/journal.pone.0022063

**Published:** 2011-07-14

**Authors:** Teresa M. Abney, Yuan Feng, Robert Pless, Ruth J. Okamoto, Guy M. Genin, Philip V. Bayly

**Affiliations:** 1 Department of Mechanical Engineering and Materials Science, Washington University in St. Louis, St. Louis, Missouri, United States of America; 2 Center for Materials Innovation, Washington University in St. Louis, St. Louis, Missouri, United States of America; 3 Department of Computer Science and Engineering, Washington University in St. Louis, St. Louis, Missouri, United States of America; 4 Department of Biomedical Engineering, Washington University in St. Louis, St. Louis, Missouri, United States of America; Harvard University, United States of America

## Abstract

Non-destructive measurement of acceleration-induced displacement fields within a closed object is a fundamental challenge. Inferences of how the brain deforms following skull impact have thus relied largely on indirect estimates and course-resolution cadaver studies. We developed a magnetic resonance technique to quantitatively identify the modes of displacement of an accelerating soft object relative to an object enclosing it, and applied it to study acceleration-induced brain deformation in human volunteers. We show that, contrary to the prevailing hypotheses of the field, the dominant mode of interaction between the brain and skull in mild head acceleration is one of sliding arrested by meninges.

## Introduction

What happens to the brain when the skull accelerates? This question is central to understanding the most common forms of mild traumatic brain injury, but it is still not completely answered [1], [2], [3], [4], [5]. While computer models with high anatomic accuracy promise insight into the brain's response to skull acceleration [6], [7], [8], [9], [10], validation of these models and identification of appropriate brain/skull boundary conditions are challenging because of difficulties in measuring accurate dynamic displacement fields in the human brain *in vivo* [7]. Our focus in this article is extraction of quantitative, information on the dynamic displacement of the brain relative to the skull in human volunteers during angular acceleration of the head.

Three classes of experimental data exist from which dynamic brain/skull mechanical interactions can be estimated. The first class consists of quasi-static data obtained during image-guided neurosurgery [7]. These data provide estimates of boundary conditions, obtained through solution of inverse problems that are combined with a computational model to update three-dimensional maps of the brain as the brain is manipulated during surgery. While these data are useful for estimating effects of distant boundaries on displacement of a tumor mass during surgery, they are not optimized for the distinct task of predicting dynamic effects of brain/skull interactions.

The second class of data is obtained through the bi-planar X-ray approach of Hardy et al. [11], [12], in which neutrally buoyant markers embedded in a cadaver head are tracked during high-speed impact between the cadaver head and a relatively rigid surface. Some insight into brain/skull boundary conditions can be gained from these data: Zou et al. [13] observed that the markers displaced as if connected to a rigid body when observed during lower levels of acceleration, and as if connected to a deformable body at higher levels of acceleration. While the observation of the brain sliding relative to the skull *in vivo* is supported by magnetic resonance (MR) imaging observations of the brain's responses to both mild acceleration and quasi-static deformations, [14], [15], [16], [17], care must be taken when extrapolating data from cadavers to humans. These data have been applied to validate computational models of the human head, but the sparse distribution of markers limits quantification of boundary conditions to uniform, averaged relations [18].

The third approach is the tagged MR approach [17], [19]. In this approach, displacement fields within the brains of human volunteers are tracked as the volunteers move their heads inside the core of a MR scanner. Displacement fields are interpreted through a modified version [19] of the harmonic phase (HARP) algorithm of Osman et al. [20], providing accurate estimates of dynamic strain fields [21]. Comparison of intracranial strain fields to solutions applying prescribed displacement boundary conditions suggests that regions of both fixation and sliding exist at the boundary between the brain and skull. However, the approach has not been used to obtain displacement fields because brain displacement is not meaningful, unless it is described relative to the skull. Estimation of the motion of the skull from MR data sets presents an additional challenge, since tagged MR images contain relatively little contrast in bony skull tissue, and adjacent soft tissue (scalp) moves relative to the skull.

In this article, we identify the dominant modes of brain displacement relative to the skull through a method based upon principal component analysis of tagged MR images. Our method involves an algorithm for aligning a series of sequential dynamic MR images to determine the rigid body motion of the skull, a HARP-based analysis to identify displacement fields in the brain relative to the skull, and principal component analysis to identify the dominant modes of displacement.

Principal component analysis and the Karhunen–Loève transform decrease the dimensionality of a data set by identifying the set of orthogonal basis vectors that describes a data set most efficiently [22]. The first principal component is the basis vector that contributes the maximum amount of variance to the dataset, and the subsequent principal components contribute successively smaller fractions of this variance. This approach has been applied to many physical and image processing problems, including face recognition [23]. The specific algorithms used in the current study were adapted from Jacobs et al. [24]. By decomposing displacement patterns from images into principal components reminiscent of dominant vibrational modes, we provide insight into the brain/skull boundary conditions appropriate for mild angular head acceleration, and the response of the brain to such loading.

In the following, the method is first demonstrated by application to a simulation of a vibrating plate with pressure uniformly and suddenly applied to one face. The method is next applied to dynamic, tagged MR images of a rotating cylindrical gelatin MR phantom. The article concludes with application of the method to displacement data obtained for human subjects undergoing angular head acceleration.

## Methods

Three steps were involved in converting sequences of dynamic MR images into principal components of displacement fields: (1) identification of rigid body translations and rotations of the skull, (2) identifying displacement fields within the images, and (3) identifying the principal components of the displacement fields. We follow a description of these protocols with descriptions of the model problems and human data to which the protocols were applied.

### 1. Estimating principal components of a displacement field from MR images

#### 1.1. Image alignment

In sequences of images for which the reference body (e.g. the skull) underwent rigid body motion relative to the image frame of reference (e.g. the MR scanner), rigid body motion of the skull was estimated and subtracted before subsequent displacement estimation. The procedure was to find the translation and rotation of each image that best aligned the reference body with its position in the first frame of the sequence.

A two-step cross-correlation technique was used on “masked images.” The deforming region of interest (e.g. the brain) within each image of the sequence was masked out by manually digitizing the boundary between the deforming region of interest and the reference body. Image data within this region of interest was replaced with a patch of uniform gray scale intensity equal to that region's mean grayscale intensity. The image alignment procedure then began with the first step: coarse rotational alignment between the first masked image in the sequence and each subsequent masked image. 360 template images were created from the first masked image by rotating the image in 1° increments and using bi-quadratic interpolation to interpolate pixel values. Cross-correlations were performed between each rotated template and each masked image in the sequence. These were done efficiently by pre-computing the discrete Fourier transform of each rotated and masked image, allowing the cross-correlation maps to be computed for all translations in a single element-wise matrix multiplication. The angle of rotation of the template with the highest peak cross-correlation value was noted as a first order approximation of rotation angle.

The second step in the procedure was a refinement to obtain sub-pixel resolution in the image alignment. 900 templates were generated within ±15° of the first order rotation angle and the cross-correlation procedure was repeated. Here, sub-pixel resolution was obtained by fitting a bi-quadratic patch to the 9 points including and surrounding the discrete cross-correlation peak, and finding the location of the interpolated peak. This refinement yielded approximately ±0.2 pixel accuracy in reproducing rigid body translations and rotations of 100x100 pixel test images that underwent known transformations.

#### 1.2. Estimation of displacement fields

Temporary magnetic “tag lines” were imposed on MR images by applying RF pulses in combination with magnetic gradients. Tagged images of a gel cylinder and brains of three human volunteers were analyzed to estimate displacement fields. Phase contours of the dominant spatial frequencies in the tagging pattern were tracked using a modification of the harmonic phase (HARP) method [20] by Bayly et al. [19]. Briefly, the phase is a property of a material point for an interval of time that exceeds the measurement interval; therefore, by tracking intersection points of phase contours, displacement fields can be estimated. Displacement vectors 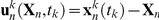 were calculated for each intersection point *n* = 1,2,…,*N* and each time step *k* = 1,2,…,*K* where 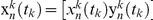 and 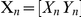 are final and initial positions of material point *n*, respectively.

#### 1.3. Principal component analysis

Principal components of the estimated displacement vectors 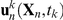 were calculated for each of the *K* time frames and at each of the *N* intersection points. A reshaped array of displacement components was defined: 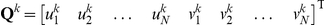, where 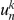 and 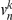 are the horizontal and vertical components, respectively, of 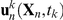, written in the coordinate frame of the first (undeformed) image of the sequence. Singular value decomposition was performed. The full 2*N*x*K* matrix of displacements at all times and locations, **Q**, was represented as:
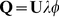
(1) where **U** is a coefficient matrix, ϕ is a 2*N*×*K* matrix of *K* eigenvectors 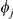 (*j* = 1, 2, …, *K*), and λ is a *K*×*K* diagonal matrix of *K* eigenvalues λ*_j_*. Each displacement array **Q** *^k^* was written as the weighted sum of eigenvectors at each time *k*:
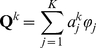
(2) where the modal coefficient 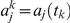 is the temporal variation representing the contribution of principal component *j* at time frame *k*. Taking the inner product of both sides of Equation (2) with the vector 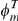 and noting that 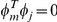 except where *m* = *j*, the modal coefficients can be written:
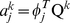
(3)


The original displacement field can be approximated using only the first *p* principal components:
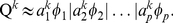
(4)


The sum of the *p* largest eigenvalues, divided by the sum of all eigenvalues, represents the fraction of the variance in the data captured by this approximation.

#### 1.4. Model problem: vibrating simply supported plate

The principal component analysis method was first studied through application to simulated images of a simply supported 2-D Kirchoff plate of dimensions *a*×*b* subjected at time *t* = 0 to a uniformly distributed constant force on one face. The response to this loading is a time-varying out-of-plane displacement 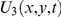 [_25_]:
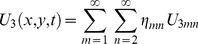
(5) where the vibrational mode shapes are:
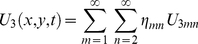
(6) and the modal coefficients are:
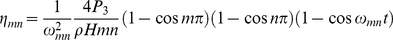
(7) for frequency
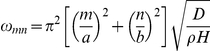
, in which *D*, *H*, and ρ are the stiffness, thickness, and density of the plate, respectively. For these boundary conditions, only modes where *m* and *n* are both odd contribute to the solution.

### 2. Experimental

#### 2.1. Angular deceleration of a gelatin cylinder

Displacement fields were estimated from dynamic MR images of a viscoelastic gelatin cylinder subjected to an angular deceleration pulse. The MR images were acquired as described elsewhere [19]. Briefly, a gelatin MR phantom was prepared in a relatively rigid cylindrical container (inner diameter 56 mm), and placed in a MR-compatible rotation device that imparted a prescribed, repeatable rotation to the specimen that was stopped by a repeatable mild impact. During the impact peak acceleration was 27.7±3.5 m/s^2^ and the duration was approximately 45 ms. Motion of the cylinder triggered a fast gradient-echo MR imaging sequence (FLASH 2D) in a 1.5T MR scanner (Sonata, Siemens, Malvern, PA). The sequence superimposed “tag lines” over the normal MR image of the cylinder [26]. These sinusoidal variations in brightness, which move with material points in the cylinder, served as non-invasive markers for tracking motion. The two orthogonal sets of tag lines were each spaced 10 mm peak-to-peak. The sequence and the cylinder rotation were repeated 144 times, with a portion of the data for images at each of *K* = 60 time-points sampled in each repetition. The resulting temporal resolution was 6 ms per frame.

#### 2.2. Angular deceleration of a human head *in vivo*

Displacement fields within the brains of human volunteers were estimated from series of dynamic MR images. The mechanical device used to rotate the gelatin cylinder was adapted to hold a volunteer's head and apply a prescribed, repeatable rotation and stopping force (additional detail in [17]). A fast gradient-echo MR sequence was triggered by motion of the volunteer's head. A series of *K = *90 tagged MR images were obtained (time resolution 6 ms) by acquiring a part of the image data during each repetition, and 12 time frames in the vicinity of the measured acceleration peak were analyzed. The imaging plane was a coronal plane parallel two centimeters above the genu and splenium of the corpus callosum. Each image had a resolution of 192×144, with a voxel size of 2 m^2^. Spatial resolution of tag lines was 8 mm. All protocols were approved by the Washington University School of Medicine Human Research Protection Office, and written informed consent was obtained from all participants. The three volunteers underwent differing peak angular accelerations: 299±29 rad/s^2^, 244±7 rad/s^2^, and 370±21 rad/s^2^ (mean±SD).

## Results

### 1. Demonstration of the method on model data

**Figure 1:**
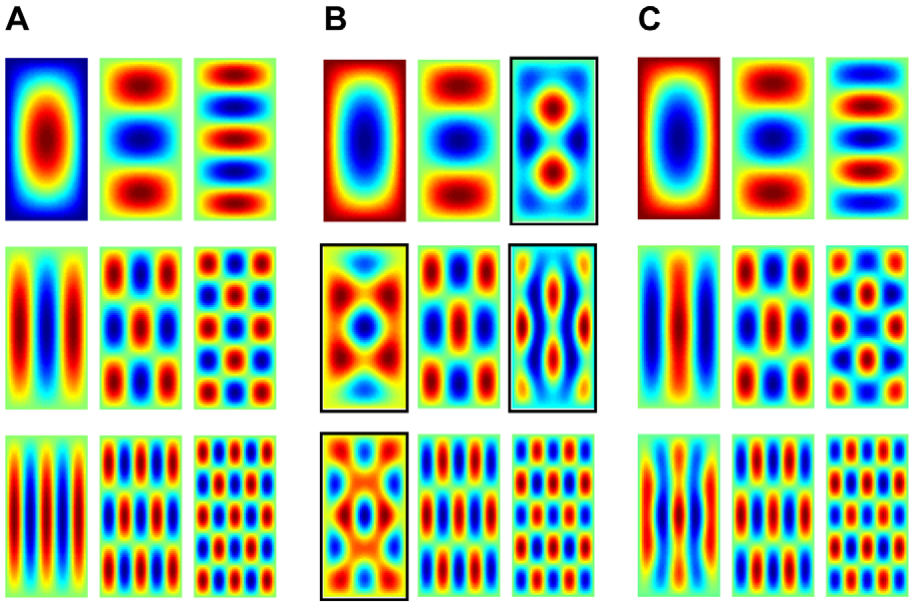
Vibrating, simply supported Kirchoff plate subjected to a uniform, instantaneously-applied, face loading. (A) Theoretical vibrational modes of a two-dimensional vibrating plate (2×1 units). Contours indicate the relative displacement out-of-plane (red positive; blue negative). (B) The first nine principal components of vibration; four of the nine differ from the “input” vibration modes used to generate the displacement field. (C) The input modes can be reconstructed from the linear combinations of the estimated principal components.

The principal component algorithm was first applied to data from a model of a simply supported Kirchoff plate subjected to a uniform, instantaneously-applied, face loading, with the goal of highlighting problems associated with deriving displacement modes from images. The plate responded to this loading with vibration in the out-of-plane direction. An approximate solution was generated using only the first nine non-zero vibrational modes in each spatial direction (Figure 1A). When principal component analysis was performed on the simulated displacements, nine mode shapes contributed to the total variance (Figure 1B). However, in cases in which the modal coefficients were degenerate (Figure S1), the mode shapes recovered from principal component analysis did not match the vibrational modes that were used as input. Degeneracy was evident when the time series of two modal coefficients exhibited similar frequencies; principal components 3 and 4 were degenerate, as were principal components 6 and 7 (Figure S1). Principal components were recovered by forming linear combinations of the degenerate modes (Figure 1C). Noise typically confounds the interpretation of the less significant principal components. In all cases, complete reconstruction of the input dataset could be achieved by combining all nine principal components, as can be seen from a plot of the cumulative fraction of total variance represented by the nine principal components (Figure S1).

### 2. Calibration of the method on an MR phantom

The complete method was then applied to quantify deformations within a cylindrical, viscoelastic MR phantom (a 10 cm core of gelatin within a relatively rigid acrylic pipe) that was rotated about its axis of axisymmetry. The additional steps required for this analysis included identifying and removing rigid body motion of the cylinder's outer boundary from the temporal sequence of MR images and assessing effects of MR noise.

**Figure 2:**
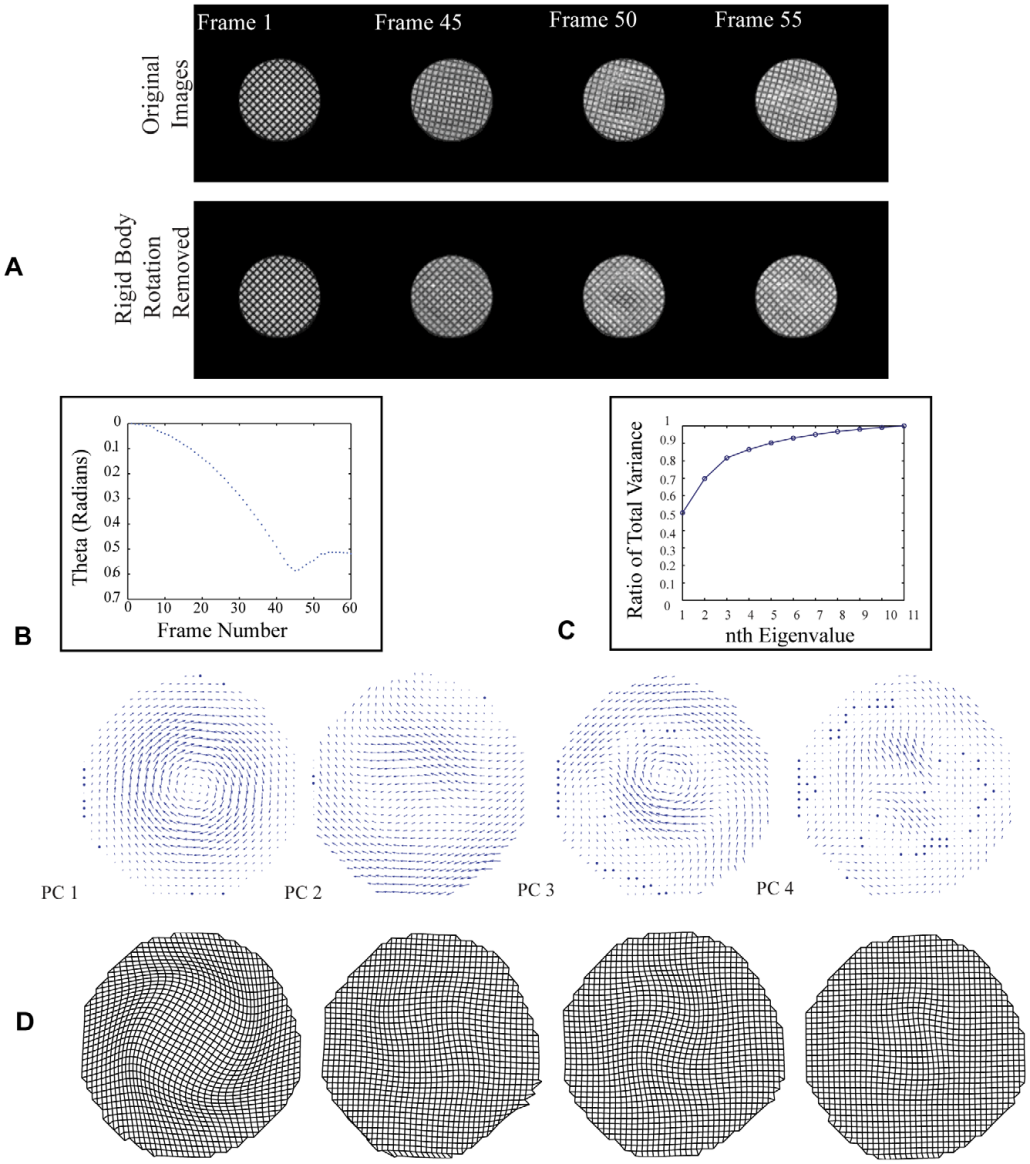
Method applied to rotating viscoelastic gelatin cylinder. (A) MR images of the gel phantom cylinder (top) were rotated to remove rigid body motion (bottom) prior to analysis. (B) The procedure resulted in a prediction of the time course of the cylinder's angular motion using the outer boundary. (C) More than 80% of the total variance is explained by the first four principal components. (D) Quiver plots for each principal component (PC), from left to right, are shown for the gel cylinder. Plots are scaled differently to highlight detail, so magnitude is not equivalent between principal components. Corresponding deformed mesh grids for the first four principal components are scaled by their eigenvalues.

A series of 60 MR images was acquired following a sudden arrest of the MR phantom's rotation, with an orthogonal grid of temporary magnetic tag lines superimposed on and deforming with the cylinder's cross-section. A set of 11 of the images near the peak deceleration pulse (frames 45–55), were analyzed to quantify the locations of tag line intersections. Rigid body rotation and translation were estimated from “masked images” in which all but the outermost 5 mm of the gelatin was masked. This rigid body motion was subtracted using a two-step cross correlation approach to emphasize displacement of the gelatin relative to that of the cylindrical container, in the same way that motion of the brain relative to the skull will be emphasized below (Figure 2A–2B).

**Figure 3:**
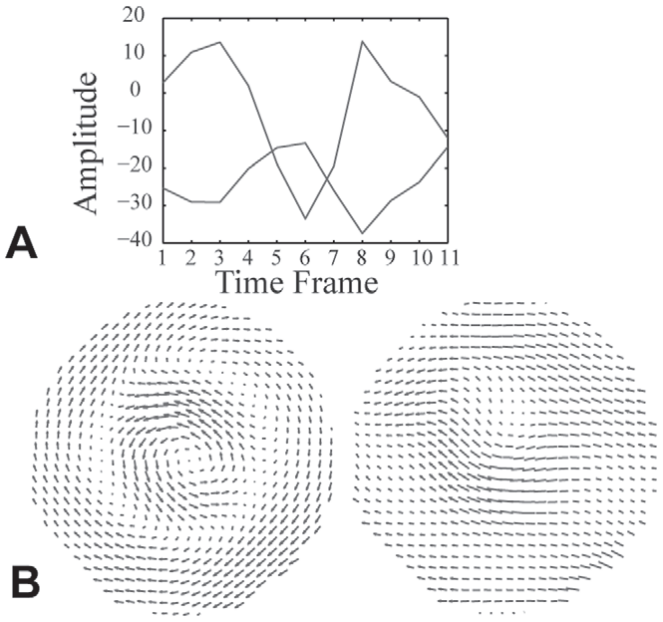
Method indicating degeneracy of principal components. (A) Modal coefficients of principal components 2 and 3 overlaid to show their similar frequency. (B) Linear combinations of principal components 2 and 3 (the difference, left, and sum, right) yield intuitive radial patterns.

Estimated displacement fields from MR images of the rotating cylinder were then analyzed using principal component analysis. The method yielded 11 non-zero principal components, but only the first few were significant contributors to the observed deformation patterns, as evidenced by their modal coefficients: the first four 4 principal components accounted for over 80% of the total variance in the dataset (Figure 2C). Although the remaining principal components were significantly smaller contributors, all 11 principal components were required for full reconstruction of the dataset. While the gelatin did not slip relative to the cylindrical container, boundary motion artifacts were evident in the less significant principal components. Displacements for each principal component in Figure 2D were scaled by that modes contribution to the total variance (Figure 2C). Modes with similar temporal variation in modal coefficients, indicating a degeneracy of modes, were combined to form alternative principal components (Figure 3A–3B) that resembled the Bessel functions predicted in the linear viscoelastic solution to this problem [21], [27]. Strain fields for the first four principal components, calculated in both Cartesian coordinates and polar coordinates (Figure S2), showed intuitive distributions as well, with shear strain fields in radial coordinates again resembling the expected Bessel functions.

### 3. Human brain *in vivo*

**Figure 4:**
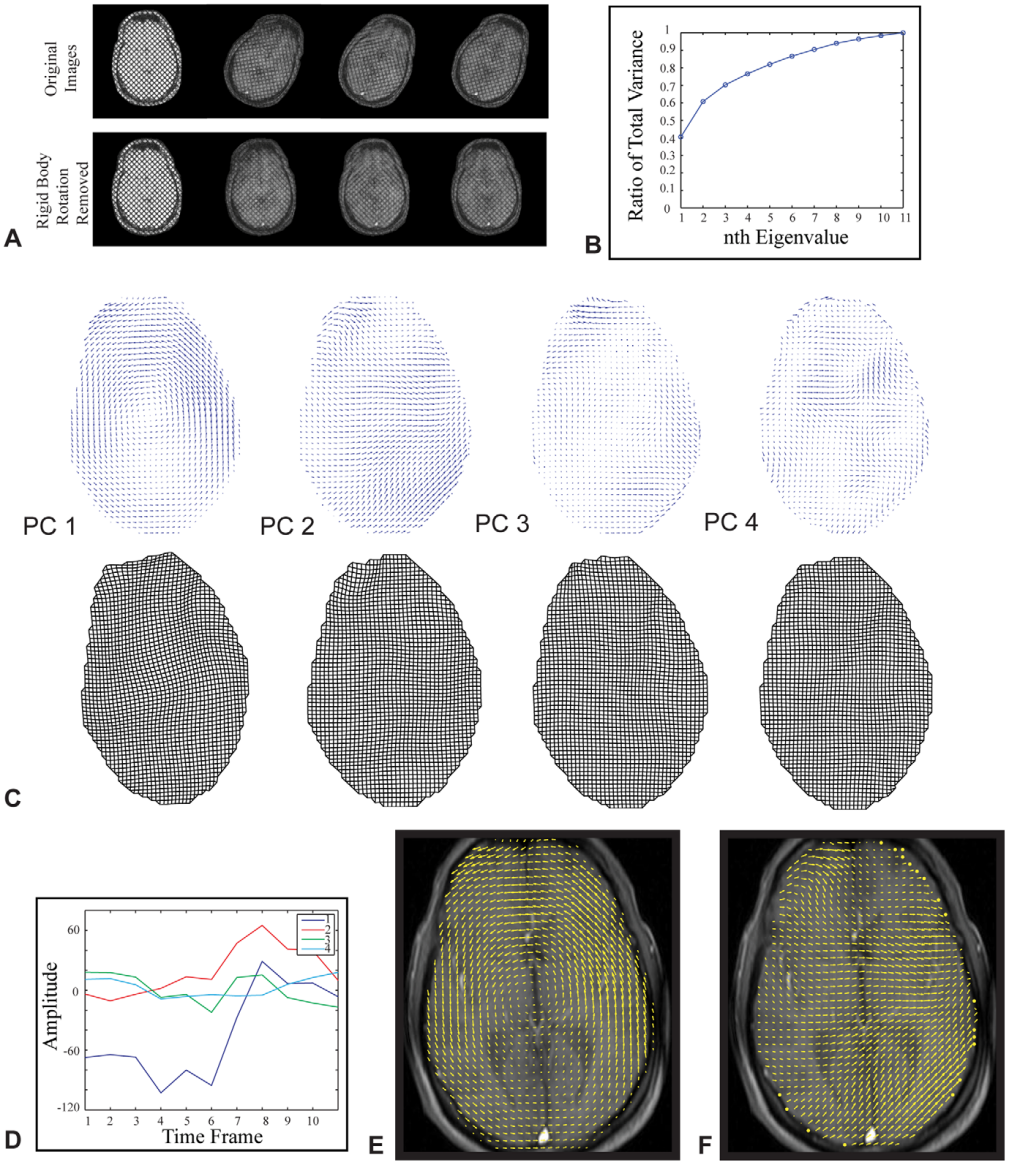
Method applied to the human brain. (A) Rigid body motion of the skull was removed prior to principal component analysis. (B) First four principal components account for approximately 75% of data set variance. (C) Quiver plot of first 4 principal components (top) associated with the mechanical response of the brain to a rotational deceleration (shown from left to right). Plots are scaled independently to highlight detail within each of the principal components. Deformed mesh grids for the first four principal components (bottom), scaled in proportion to their eigenvalues. (D) Modal coefficients show no evidence of degeneracy indicated by unique temporal variation. (E) PC1 overlaid on a scout image to emphasize anatomical correlation. (F) PC2 overlaid on a scout image.

A 90 time-frame MR image set was acquired during the angular deceleration of a human head, with tag lines superimposed and deforming with the tissue (Figure 4A). To emphasize brain/skull interactions, rigid body translation and rotation were first subtracted using the two-step cross-correlation approach. As with the gelatin cylinder, analysis of 11 images relative to the undeformed image yielded 11 non-zero principal components. Boundary effects were again evident only in the principal components with modal coefficients having lower peak amplitude (Figure 4C: vector plots are scaled independently, while deformed grid plots were scaled in proportion to their modal coefficients). Approximately 75% of the total variance in the images was explained by the first four principal components. As with the gelatin cylinder, although the remaining principal components were smaller contributors to the total variance, all 11 principal components must be combined for a full reconstruction of the dataset (Figure 4B).

## Discussion

We address here two aspects of the method–the importance of sub-pixel resolution and the limitation of boundary artifacts–and then discuss the observations about relative motion of the brain and skull in the context of these limitations.

### 1. Sub-pixel resolution is achievable in alignment of MR images

The first step towards applying the method to brain/skull displacements was removing the effect of rigid body rotations, which otherwise would have dominated principal components. In the case of the rotating cylindrical MR phantom, the gelatin core did not slip relative to the plastic pipe that encased it. After the rigid body correction, this boundary condition was evident from the results of the principal component analysis: very low displacements were evident at the outer boundaries of each of the most important mode shapes.

### 2. Boundary artifacts appear in principal components with lower modal coefficients

In the case of the gelatin cylinder, the first principal component, which represented the most dominant mode of displacement in the cylinder and comprised over half of the total variance, had displacements that were nearly zero on the outer boundary. The second and third principal components were not symmetric, but had similar temporal variations in their modal coefficients (Figure 3A); they were degenerate modes that, like those of the vibrating plate, could be combined to yield a mode shape that was more intuitive (Figure 3B). Note that each principal component quiver plot was auto-scaled to show detail and thus magnitudes cannot be compared throughout the sequence pictured (Figure 2D).

The first four principal components reflect expected features of the displacement field. Beyond the fourth principal component, artifacts appeared at the outer boundary. When intersection points are determined using the HARP method, measurement error is expected to be greatest at the outer boundary [21]. Consequently, artifacts in the form of boundary effects overpowered the higher principal components.

### 3. The dominant mode of brain displacement is one of sliding relative to the skull

Principal component analysis shows that the dominant mode of displacement was one of rotation of the brain relative to the skull for the individuals tested. The first principal component accounted for about 40 percent of the total variance (Figure 4B). This first principal component of brain displacement differed from that of the gel cylinder in two important ways: first, the brain clearly slides relative to the skull, while the gel did not slide relative to its outer shell; second, the brain's displacement was non-symmetric, with the anterior and posterior regions having significantly different contributions to the first mode, while the gel exhibited axisymmetry. This was evident as in strain fields associated with the first four principal components (Figure S3).

As with the gelatin cylinder, the first four modes accounted for the majority of the variance. Modes beyond the fourth mode are expected to have low signal to noise ratio; what information they do contain can be expected to have little influence on the overall response. The second principal component presents a displacement field largely in the direction of impact, radiating from the impact point. The third and fourth principal components present predominantly interior vibrational modes, possibly related to relative motion of anatomical structures. The temporal variations of the modal coefficients for these modes appeared to be independent, suggesting that modal degeneracy is not a factor in this analysis (Figure 4D). Error caused by boundary effects was again more evident in the higher modes.

**Figure 5:**
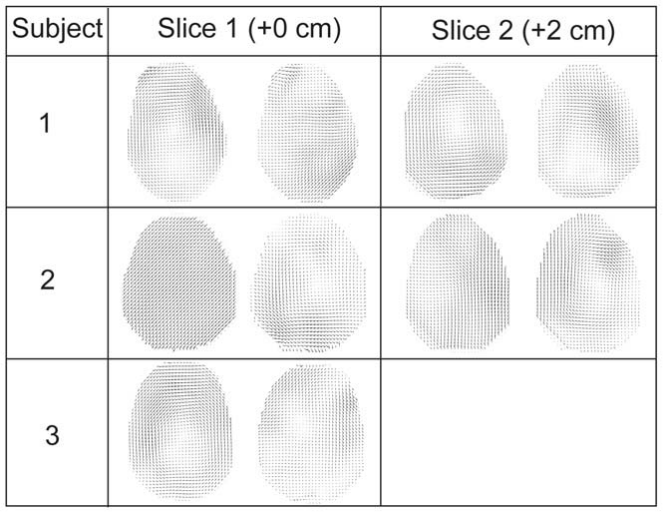
Comparison of multiple results. A subject comparison of 3 subjects in two planes 2 cm apart.

Figures 4E–4F display the first two principal components of subject 1 overlaid on a scout image to correlate displacements with anatomical features. Alternating tensile and compressive components in the displacement field associated with the first principal component are suggestive of effects of internal meninges. With boundary conditions taken into consideration, these results suggest strongly that anatomical features, specifically meninges and vessels may be important factors in the brain's mechanical response to mild acceleration, and thus in mild traumatic brain injury. Discontinuities were evident in the regions of the falx cerebri and tentorium membranes indicating an important mechanical role for these. The method produced similar results for three different subjects in two separate slices (Figure 5).

### 4. Conclusions

The MR method presented was used to decompose a set of displacement fields into an optimal orthogonal set of basis vectors. This method provides understanding of the dynamics of brain displacement during head accelerations by interpreting displacement fields in the context of the most significant modes of displacement.

The first principal components revealed the dominant modes of the displacement fields, but a limitation is that principal components can also be linear combinations of these modes. The indicator of degeneracy was a similar temporal variation of corresponding modal coefficients.

A limitation of the study is that the HARP method is least accurate at the outermost boundary of an analyzed object, where the data are most useful. As a frequency-domain technique, HARP suffers from the implicit assumption that tagging pattern extends periodically outside the image domain. HARP intersection points determined at the boundary are less reliable than those on the interior of the brain. This feature is most evident in the outer boundaries of the most minor principal components. The technique is fundamentally limited by the temporal (6 ms) and spatial (2 mm) (resolution of the images; the resolution of displacement fields is further limited by the spacing between tag lines (8 mm).

Results indicated a dominant role of sliding between the skull and brain in the response of the brain to angular deceleration about the spinal axis, with restraining effects of the internal suspension of the brain including the falx cerebri and tentorium membranes. The analysis of experimental data presented here will contribute to the development of trustworthy computer models with appropriate skull-brain interactions.

## Supporting Information

Figure S1 **Vibrating plate modal coefficients.** Modal coefficients for each principal component of the vibrating plate. The inset shows that the majority of variance was due to the first principal component. The noise evident arose because the simulation was discrete and not continuous.
(TIF)

Figure S2 **Gelatin cylinder strain plots.** Lagrangian strain fields for the first four principal components (PCs) of a rotating gelatin cylinder. The polar strain plots resemble the Bessel functions that appear in solutions to analogous problems.
(TIF)

Figure S3 **Human brain strain plots.** Lagrangian strain fields corresponding to the first four principal components (PCs) of a human brain rotating inside of a skull *in vivo*.
(TIF)
