# Supplementary figures and images for: Principal Component Analysis of Dynamic Relative Displacement Fields Estimated from MR Images
Source: PLoS One. 2011 Jul 14;6(7):e22063. doi: 10.1371/journal.pone.0022063 (PMC3136495; doi:10.1371/journal.pone.0022063)

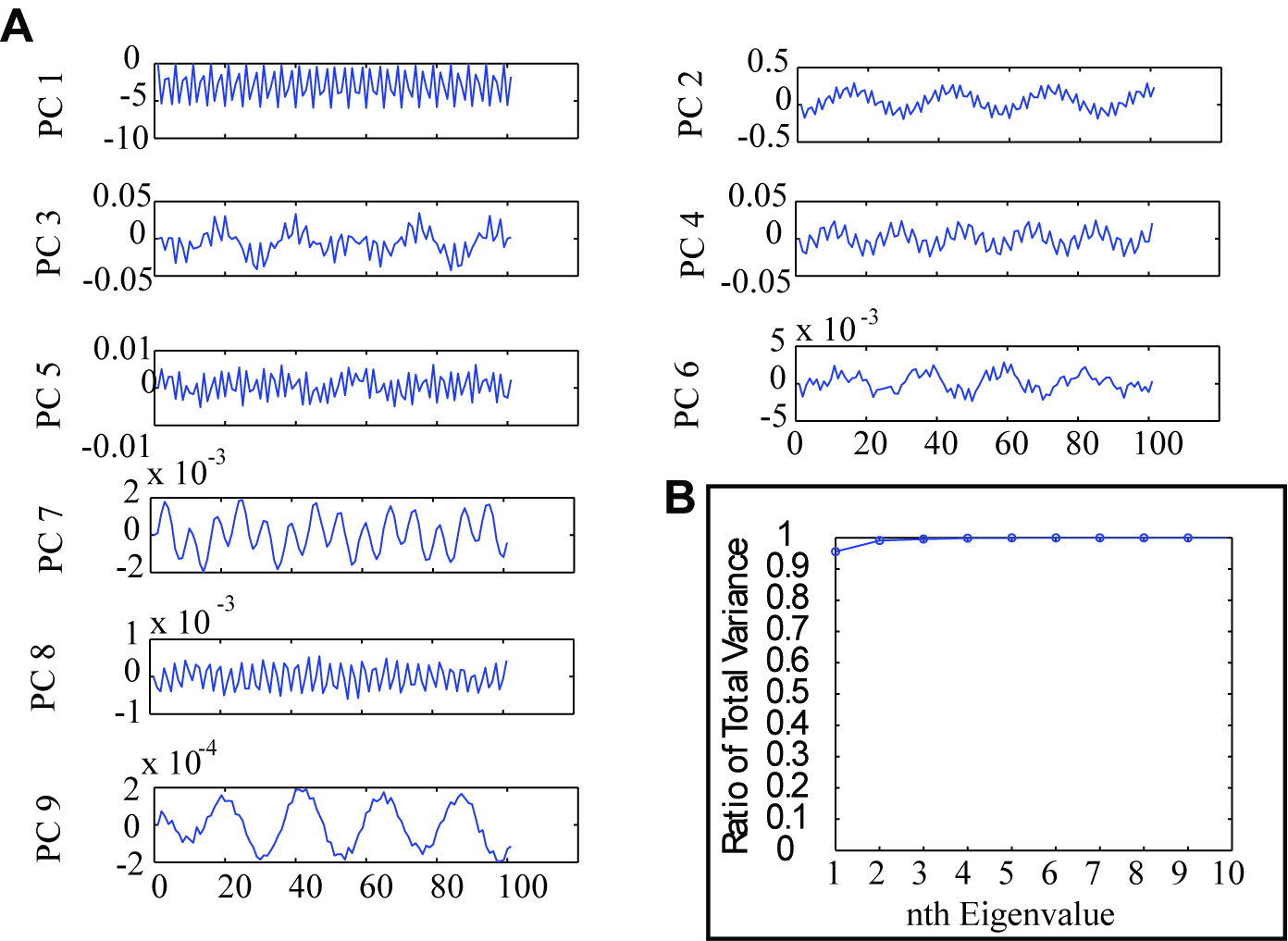

Supplement: Figure S1 — Vibrating plate modal coefficients. Modal coefficients for each principal component of the vibrating plate. The inset shows that the majority of variance was due to the first principal component. The noise evident arose because the simulation was discrete and not continuous. (TIF) [file pone.0022063.s001.tif]

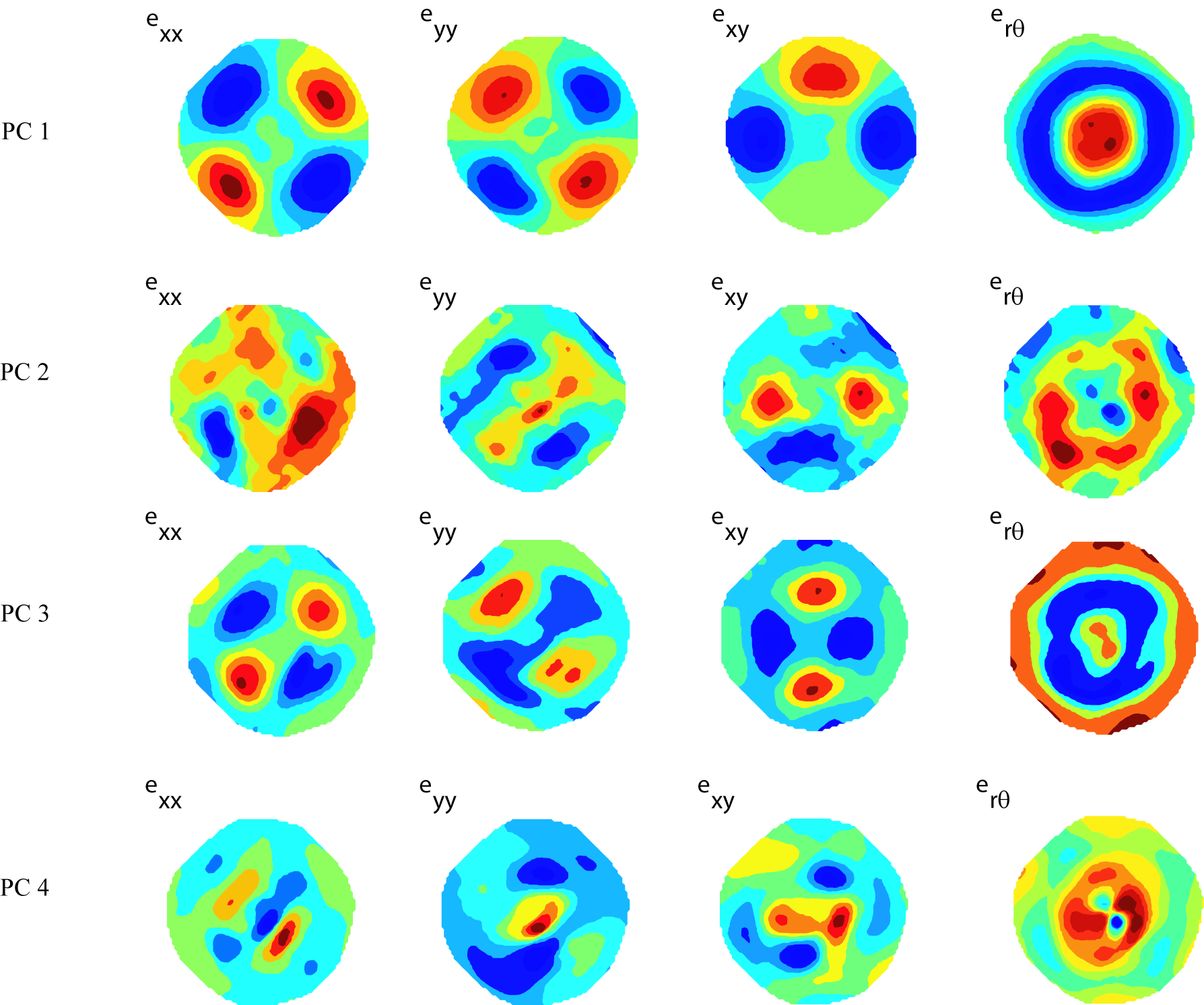

Supplement: Figure S2 — Gelatin cylinder strain plots. Lagrangian strain fields for the first four principal components (PCs) of a rotating gelatin cylinder. The polar strain plots resemble the Bessel functions that appear in solutions to analogous problems. (TIF) [file pone.0022063.s002.tif]

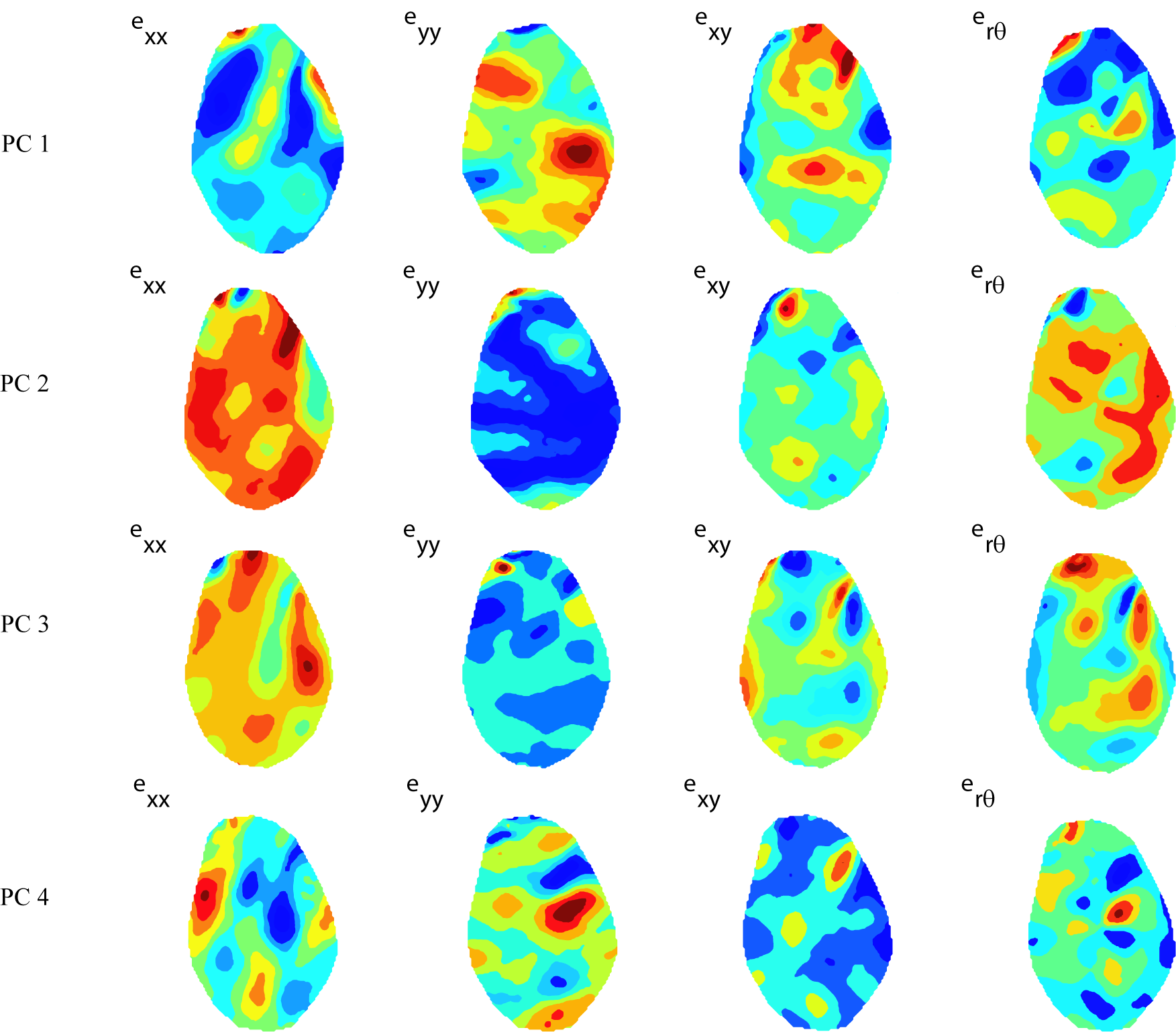

Supplement: Figure S3 — Human brain strain plots. Lagrangian strain fields corresponding to the first four principal components (PCs) of a human brain rotating inside of a skull in vivo. (TIF) [file pone.0022063.s003.tif]
